# Effects of fluid overload on heart rate variability in chronic kidney disease patients on hemodialysis

**DOI:** 10.1186/1471-2369-15-26

**Published:** 2014-02-04

**Authors:** Manuela Ferrario, Ulrich Moissl, Francesco Garzotto, Dinna N Cruz, Anna Clementi, Alessandra Brendolan, Ciro Tetta, Emanuele Gatti, Maria G Signorini, Sergio Cerutti, Claudio Ronco

**Affiliations:** 1 Department of Electronics, Information and Bioengineering (DEIB), Politecnico di Milano, P.zza Leonardo da Vinci 32, Milano, Italy; 2 Fresenius Medical Care R&D, Daimlerstrasse 15, D-61352 Bad Homburg, Germany; 3 San Bortolo Hospital, viale Rodolfi 37, 36100 Vicenza, Italy

**Keywords:** Autonomic nervous system, Fluid overload, Heart rate variability, Hemodialysis, Whole-body bioimpedance spectroscopy

## Abstract

**Background:**

While fluid overload (FO) and alterations in the autonomic nervous system (ANS) such as hypersympathetic activity, are known risk factors for cardiovascular morbidity and mortality in patients on chronic hemodialysis (HD), their relationship has not been thoroughly studied.

**Methods:**

In this observational study involving 69 patients on chronic HD, FO was assessed by whole body bioimpedance measurements before the midweek HD session and ANS activity reflected by Heart Rate Variability (HRV) was measured using 24-hour Holter electrocardiogram recordings starting before the same HD treatment. In total, 13 different HRV indices were analyzed, comprising a mixture of time domain, frequency domain and complexity parameters. A correlation analysis was performed between the HRV indices and hydration status indices. Successively, patients were retrospectively assigned to a high FO (H, FO > 2.5 L) or low FO (L, FO ≤ 2.5 L) group and these were further compared also after stratification by diabetes mellitus. Finally, a small number of patients without diabetes with significant and persistent FO were followed up for 3 months post-study to investigate how normalization of fluid status affects HRV.

**Results:**

SDANN, VLF, LZC and HF% parameters significantly correlate with FO (correlation coefficients were respectively r = –0.40, r = –0.37, r = –0.28 and r = 0.26, p-value < 0.05). Furthermore, LF% and LF/HF were inversely correlated with hydration status (correlation coefficients were respectively r = –0.31 and r = -0.33, p-value < 0.05). These results indicate an association between FO and reduced HRV, higher parasympathetic activation and reduced sympathetic response to the HD session. Indeed, group H tended to have lower values of SDANN, VLF and LZC, and higher values of HF% than patients in the L group. Finally, there was a trend towards lower LF% measured during the last 30 minutes of HD for the H group versus the L group. Reduction in FO achieved over 3 months by implementation of a strict fluid management plan resulted in an increase of HRV.

**Conclusions:**

Our results suggest that depressed HRV is associated with fluid overload and that normalization of hydration status is accompanied by improved HRV.

## Background

End stage renal disease (ESRD) patients are known to have a higher risk of mortality than the healthy population. Cardiovascular events are a major cause of this high mortality, more than 40% of all patients with ESRD die of cardiac causes [1]. Fluid overload (FO) is common in patients on maintenance hemodialysis (HD) and it can lead to hypertension, left ventricular hypertrophy and cardiac mortality [2]. Even though the genesis of LVH is multifactorial, hypertension and FO are prominent factors of particular relevance for patients on HD [3]. An abnormal hydration state has been related not only to LVH and hypertension, but also to dialysis related hypotension, pulmonary and peripheral edema, heart failure and other adverse cardiovascular events [4-6]. Moreover, FO has also been highlighted as a key independent predictor of mortality in patients on HD [7].

Alterations in the autonomic nervous system (ANS) are also associated with cardiovascular death [8,9], e.g. autonomic neuropathy in patients with diabetes and chronic kidney disease (CKD) [10]. Sympathetic overactivity and parasympathetic blunting have been observed in patients with CKD and ESRD [11,12]. This sympatho-vagal imbalance contributes to the increased risk of cardiac death in these populations [10]. Heart rate variability (HRV), which reflects the ability of the sinoatrial node to change heart rate in response to sympathetic and parasympathetic inputs, is a valuable tool for assessing heart rate control and also allows quantification of autonomic activity [10]. For instance, the spectral decomposition of HRV signals has been proved to provide valuable indices: the high frequency (HF) power reflects vagal and respiratory mediated changes in heart rate, whereas low frequency (LF) power is mostly a marker of sympathetic modulation [13].

Although alterations of the ANS and high FO are each recognized risk factors for increased cardiovascular mortality in patients on HD, the effects of fluid overload on the ANS in the ESRD population have not yet been fully elucidated.

The effects of changes in central volumes on ANS are of great interest also from a physiological point of view and many studies have been performed on this topic [14,15]. The hypothesis of the work is based on the fact that autonomic nervous system (ANS) is known to be affected by variations of central volumes as it proved from different physiological studies, e.g. protocols of lower body negative pressure (LBNP) [16], rest-tilt [17] and head down tilt bed rest [18]. In fact, a critical decrease in left ventricular filling, due to the decreased venous return, induce a sympathetic activation mediated by cardiopulmonary baroreceptor [19], and the sympathetic activations is also influenced by the hydration status [15].

The aim of this study is to investigate the relationship between fluid overload and alterations of the ANS in patients on chronic HD using FO measurements from the Body Composition Monitor (BCM, Fresenius Medical Care) and using indices of HRV as markers for ANS activity. Furthermore, as diabetes has a negative effect on the ANS, results for patients with and without diabetes are also analyzed separately. A small group of non-diabetic patients with persistent and high FO was selected for studying the effect of reducing FO on HRV over a 3 month follow-up period, the objective is to observe if ANS markers provided by HRV analyses can be modified and improved by reducing FO, in particular to observe if the low HRV and high sympathetic activity can be reversed by reducing FO.

## Methods

### Study design & setting

Eighty patients on chronic HD receiving treatment at the San Bortolo Hospital, Vicenza, Italy were enrolled in this observational study between April 1, 2009, and October 30, 2009.

### Participants

Inclusion criteria were age above 18 years and HD vintage of at least 6 months. Exclusion criteria were a HD frequency other than thrice weekly and hospitalization or antibiotic treatments in the preceding 8 weeks. The Institutional Review Board of San Bortolo Hospital approved the study. Patients signed informed consent prior to enrollment and the study was conducted in full accordance with the Declaration of Helsinki.

### Clinical data and study size

**Figure 1:**
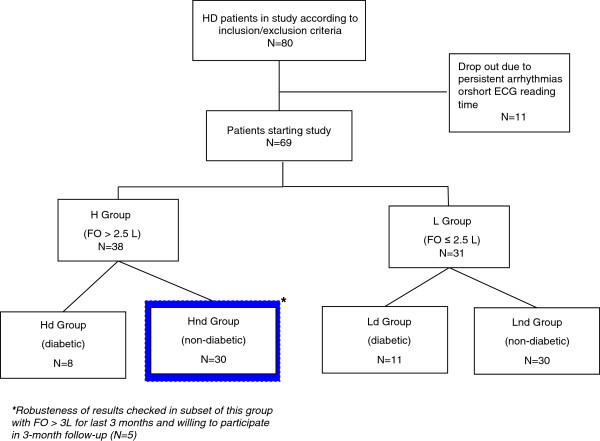
Study flow chart.

Whole body bioimpedance spectroscopy was done just before the midweek HD session. 24 hour-ECG Holter was started prior to the same midweek HD. The patients were retrospectively classified by degree of FO into two groups: group H encompassed patients with FO > 2.5 L (high FO) and group L encompassed patients with FO ≤ 2.5 L (low FO). This threshold was chosen based on studies indicating an increased mortality in patients with FO > 2.5 L [20,21]. H and L groups were successively compared after stratification by diabetes mellitus. Four groups were thus considered: diabetics with high (Hd) and low FO (Ld), non- diabetics with high (Hnd) and low FO (Lnd). Figure 1 provides an overview of the study design and the patient numbers in each of the four groups.

The investigation results were subsequently tested by exploring if reductions in FO can favorably influence HRV. To this end, a small number of non-diabetic patients (5) with significant and persistent FO (i.e. FO > 3 L in the last 3 months) and who signed informed consent were enrolled in a subsequent 3-month fluid management plan from October 2010 and May 2011. A FO of < 2.5 L was targeted for the end of the first month; this level was then maintained in the two successive months. 24 hr ECG Holter recordings were performed, as described before, at the start of the study (baseline, BL), after 1 month (1 M), when the target was supposed to be reached, and after 3 months (3 M).

No changes in medication and drug prescription were made during the study or during the 3-month post-study follow-up.

### Measurements

To determine the hydration state, clinical surrogate parameters are always considered, such as interdialytic weight gain, ultrafiltration rate or blood pressure, or concepts are subjectively invoked to define normal hydration, e.g. the achievement of target dry weight without hypotensive episodes or interdialytic hypertension. Here, a whole body bioimpedance spectroscopy method was employed. Before each HD treatment, FO was assessed by the Body Composition Monitor (BCM, Fresenius Medical Care, Germany). This measures the impedance between 5 and 1000 kHz. High-frequency current passes through the total body water (TBW), whereas low-frequency current cannot penetrate cell membranes and thus flows exclusively through the extracellular water (ECW). ECW consists of the interstitial water, the plasma water and the transcellular water. Fluid status is defined in terms of excess extracellular water using physiologic models [22,23]. The BCM provides the FO expressed in liters [7].

In addition to absolute FO just before the HD session (FO_pre_), an additional parameter was measured: FO normalized over the extracellular water (FO_pre_/ECW%). FO_pre_/ECW% facilitates comparison between patients, which may have the same FO value but a completely different distribution of fluids; in particular this index is thought to reflect the extent of central volumes accumulation.

Twenty four-hour electrocardiogram (ECG) Holter recordings were started before the HD treatment employing a three-lead Holter device with a sampling rate of 250 Hz (clickholter, Cardioline, et medical devices SpA, Italy). Eleven patients dropped out of the study for reasons related to the HRV analysis: presence of persistent arrhythmias during HD, or duration of recording shorter than the HD session for technical reasons, e.g. detachment of the electrodes.

In addition, systolic blood pressure (SBP), diastolic blood pressure (DBP), pulse pressure (PP), and FO were collected during the post-study three-month follow up involving 5 patients. These parameters were recorded during the HD treatments in the same week of ECG recording and the average values were considered for the analysis. During first month, FO reduction was performed by decreasing the post-dialysis weight treatment-by-treatment in steps defined by the attending nephrologist. The post-dialysis fluid status was assessed by subtracting the intradialytic weight loss from the pre-dialysis FO measurement. The time-averaged fluid overload (TAFO) was calculated as the mean between the pre-dialysis and the post-dialysis fluid status, thus assuming a linear accumulation of fluid in the interdialytic period. Weekly targeted post-dialysis weight was defined individually so that it translated to a TAFO of 0.8 L, which is an empirical threshold estimated from several dialysis units, and considered reachable and tolerated for all patients, even thought post-dialysis FO values can be negative. Thus, the FO was reduced according to the target of < 2.5 L after one month and this level was then maintained in the two successive months.

### HRV analysis

HRV refers to the variability of the duration between two consecutive R peaks in electrocardiograms. The R peaks were automatically identified and classified from the Holter ECG recordings (CubeHolter, Cardioline, et medical devices SpA, Italy). The obtained RR time series was subdivided into 5-minute epochs. Epochs with at least 85% of qualified sinus beats were considered and the so-called normal-to-normal (NN) intervals, i.e. all intervals between adjacent R peaks resulting from sinus node depolarization, were analyzed [24]. Mean values of 13 time domain, frequency domain and complexity indices were calculated for the first 30 minutes and last 30 minutes of HD, and for the entire HD session, to represent the initial response of the patient to HD, the final condition and an overall response during treatment, respectively. The time domain parameters were: mean NN intervals, the standard deviation of the NN intervals (SDNN), the standard deviation of the averages of NN intervals in all 5-minute segments of the recording (SDANN). The frequency domain parameters were: autoregressive power spectral analysis in the very low frequency (VLF, 0.003 < f ≤ 0.04 Hz), the low frequency (LF, 0.04 < f ≤ 0.15 Hz) and the high frequency (HF, 0.15 < f ≤ 0.4 Hz) bands, the LF/HF ratio and the normalized power LF% calculated as LF/(total power – VLF) × 100 and the normalized power HF% calculated as HF/(total power – VLF) × 100 [24]. Regarding complexity domain parameters, for each 5-minute epoch, the complexity of the signals was estimated by the Lempel Ziv complexity (LZC) after the RR time series was encoded into a string. In this work binary and ternary coding rules were adopted and the coding factor *p* was 0.01 and 0.05. Full details for the computation are described in [25].

### Statistical analyses

Lilliefors test was used to test that data come from a normally distributed population. According to the test for normal distribution, the correlation between the HRV indices and FO parameters was analyzed by the Pearson or Spearman rank test, respectively. The same analyses were repeated by separating diabetic from non-diabetic patients. The comparison between the HRV indices of the two groups H and L was performed by Student’s test or Mann–Whitney–Wilcoxon test. Categorical variables were expressed as number, and comparisons between groups were performed using Fisher’s Exact test. When the two groups were subdivided into two subsets each according to diabetes (Hd and Hnd, Ld and Lnd), one-way ANOVA was performed to identify differences between groups and post-hoc comparisons were performed by Tukey’s least significant difference (LSD) test.

In the post-study 3-month results-exploratory phase, HRV indices, FO and blood pressure data were analyzed by one-way ANOVA for repeated measurements. Comparisons with baseline values were performed in a paired fashion, by means of the Wilcoxon signed rank test and the Student test and by using Bonferroni correction.

Continuous variables are expressed as means ± standard deviation, or as medians with 25° and 75° percentiles. A p-value < 0.05, 2-tailed, was considered statistically significant.

## Results

### Participants & descriptive data

A complete set of Holter recordings and reliable BCM measurements were available for 69 patients who completed the study (Figure 1). Table 1 reports the data for the two groups of patients: patients with high FO (group H, FO_pre_ >2.5 L) and with lower FO (group L, FO_pre_ ≤2.5 L). Notice that no severely obese or severely underweight patients are present in the current study (see BMI in Table 1). The patients enrolled in this study were pretty stable and their clinical history did not include hypotension proneness. We reported also the HD related events, such as cramps or dizziness, experienced by the patients after the dialysis treatment only, so did not affect the treatment administration. Patients in group L reported a shorter HD vintage compared to group H only. Sixty-seven patients (97%) were classified as hypertensive according to drug prescription and/or blood pressure values.

**Table 1:** Patient data

	**FO**_**pre**_ **≤ 2.5 L (L group)**	**FO**_**pre**_ **> 2.5 L (H group)**	**P**
**N**	31	38	
**FO [L]**	**1.1 (0.55, 1.6)**	**3.4 (2.8, 4.4)**	**<0.001**
**FO/ECW%**	**7.0 (3.6, 9.3)**	**17.7 (15.7, 25.3)**	**<0.001**
**Diabetes**	11	8	0.2
**Age [years]**	65 (50,75)	69 (60, 75)	0.2
**Gender [m/f]**	18/13	27/11	0.3
**Dialysis vintage [years]**	**4 (2, 7)**	**7 (5, 11)**	**0.02**
**Patients with residual renal function**	0	1	0.9
**Treatment time [min]**	236 (233, 241)	237 (234, 241)	0.5
**UFR [L/hr]**	0.67 (0.47, 0.84)	0.81 (0.56, 0.9)	0.2
**UFR [ml/kg BW/h]**	9.5 (5.9, 10.9)	10.9 (8.0, 13.6)	0.1
**Dry body weight [kg]**	74.2 (61.6, 78.6)	68.4 (61.0, 74.5)	0.2
**BMI**	26.4 (23.5, 28.8)	24.3 (21.6, 26.7)	0.2
**Treatment modality [HDF-OL/HD/HFD/HF]**	3/26/2/0	6/27/3/2	0.6
**HD related episodes (cramps, dizziness, hypotension)**	5	6	0.9
**Left ventricular hypertrophy**	21	25	0.9
**Peripheral vascular disease**	17	12	0.8
**Coronary heart disease**	10	11	0.2
**Hypertension**	31	36	0.5
**β-blockers**	10	14	0.8
**ACE inhibitors**	9	11	0.8
**Calcium antagonist**	14	16	0.9

The values are expressed as median (25°, 75° percentiles) or numbers of patients.

HDF-OL: Hemodiafiltration OnLine; HD:Hemodialysis; HFD: High Flux Dialysis; HF: Hemofiltration.

Significant differences are indicated in bold.

### Correlation analysis and comparisons of patients stratified according to FO

**Figure 2:**
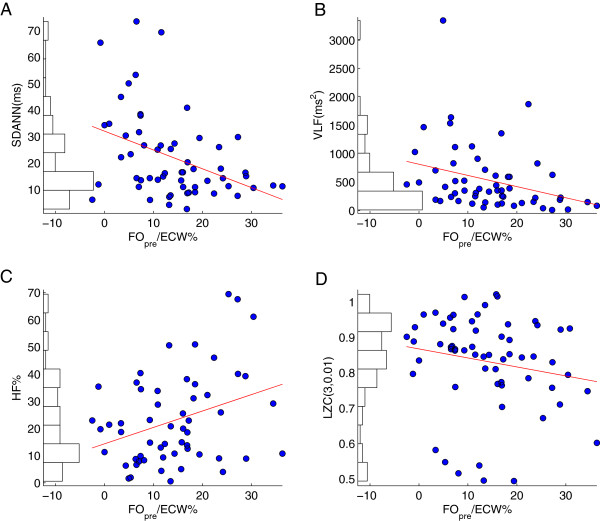
**HRV indices that significantly correlated with FO/ECW% index during the first 30 minutes of HD treatment.** Figures **A**, **B**, **C** and **D** depict the results of the correlation analysis with the regression lines for all 69 patients for SDANN, VLF, HF% and LZC (3,0.01) indices, and the correlation coefficients were r = –0.39, r = –0.39, r = 0.29 and r = –0.26, respectively. The histograms of HRV parameter distributions are shown on the left of each panel.

Analysis of the correlation between all 13 HRV indices and the two FO parameters was conducted. For clarity, only those correlations yielding statistically significant results are presented in Table 2 for the 69 patients. HRV estimated during the all three times (i.e. first 30 mins, last 30 mins and entire HD session) showed a significant negative correlation (SDANN, VLF and LZC parameters) with hydration status parameters, while a significant positive correlation (HF%) was observed for the first 30 mins and the entire HD session. Furthermore, LF% and LF/HF were inversely correlated with the FO/ECW% for the last 30 mins of HD (Figure 2). The magnitudes of these HRV values and those for the entire HD session are provided in Table 3, as are the respective values according to FO group and according to diabetes status. In general, patients in group H tended to have lower values of SDANN, VLF and LZC, and higher values of HF% than patients in the L group, whereby the differences for SDANN reached statistical significance for Hd versus Ld and for Hnd versus Lnd. Finally, there was a trend towards lower LF% measured during the last 30 minutes of HD for the H group versus the L group (L group: LF% = 42 (29,54); H group: LF% = 32 (17,50), p-value = 0.07).

**Table 2:** Correlation coefficients

	**First 30′ HD**		**Last 30′ HD**		**Entire HD**	
	**FO**	**FO/ECW%**	**FO**	**FO/ECW%**	**FO**	**FO/ECW%**
**SDANN**	-0.40^*^	-0.39^*^	**-0.29**^**#**^	-0.35^*^	**-0.31**^**#**^	-0.30^*^
**VLF**	-0.37^*^	-0.39^*^	-0.25	**-0.27**^**#**^	**-0.25**^**#**^	-0.28^*^
**LF%**	-0.09	-0.11	**-0.33**^**#**^	**-0.37**^**†**^	-0.17	-0.16
**HF%**	**0.28**^**#**^	**0.29**^**#**^	0.13	0.17	0.19	**0.30**^**§**^
**LF/HF**	-0.21	-0.22	**-0.31**^**#**^	**-0.34**^**#**^	-0.22	-0.22
**LZC (2,0.01)**	-0.18	-0.17	-0.17	-0.2	-0.13	-0.23
**LZC (2,0.05)**	-0.23	**-0.26**^**§**^	-0.15	**-0.30**^**§**^	-0.07	-0.1
**LZC (3,0.01)**	**-0.26**^**#**^	**-0.26**^**#**^	-0.34	**-0.31**^**#**^	**-0.29**^**#**^	**-0.27**^**#**^

Correlation coefficients between the hydration status parameters and those HRV indices that reached statistical significance for all 69 patients enrolled in the study. Significant values are indicated in bold.

^*^Spearman p-value < 0.01, ^#^p-value < 0.05, ^§^Pearson p-value < 0.05.

**Table 3:** Values of HRV indices for the two groups of patients

		**L group**	**H group**	**Mann–Whitney Wilcoxon**	**Ld**	**Hd**	**Lnd**	**Hnd**	**One-way ANOVA**
**# patients**		**31**	**38**		**11**	**8**	**20**	**30**	
**First 30′HD**									
	**SDANN (ms)**	**27(14,38)**	**13(9,17)**	**P = 0.001**	14(10,41)	14(9,17)	29(22,38)*	**13(10,17)**	**P = 0.002**
	**VLF (ms**^**2**^**)**	**460(291,1052)**	**227(122,594)**	**P = 0.01**	310(223,477)	124(44,300)	614(375,1123)	252(141,622)	P = 0.1
	**HF%**	**14.8(8.2,23.0)**	**25.5(11.2,39.0)**	**P = 0.04**	23.0(7.4,35.8)	28.8(22.0,53.6)	12.1(8.3,20.6)	21.4(9.9,38.7)	P = 0.03
	**LF%**	37.3(25.0,50.0)	43.0(28.6,52.3)	P = 0.4	34.3(23.9,40.7)	36.0(30.4,46.1)	43.0(28.6,52.3)	33.1(23.7,45.1)	P = 0.7
	**LF/HF**	2.72(1.44,5.17)	2.81(1.72,5.78)	P = 0.1	1.55(0.83,3.11)	1.53(0.61,2.12)	2.81(1.72,5.78)	2.04(0.87,3.41)	P = 0.8
	**LZC (3,0.001)**	0.88(0.85,0.94)	0.83(0.76,0.91)	P = 0.07	0.87(0.86,0.92)	0.81(0.77,0.92)	0.88(0.84,0.95)	0.84(0.74,0.91)	P = 0.6
**Last 30′ HD**									
	**SDANN (ms)**	17(13,27)	20(13,35)	P = 0.1	15(13,21)	12(10,16)	20(13,35)	13(9,31)	P = 0.3
	**VLF (ms**^**2**^**)**	634(394,2082)	795(459,2167)	P = 0.2	504(372,671)	246(30,860)	795(459,2167)	460(202,1645)	P = 0.6
	**HF%**	12.41(9.04,20.57)	11.41(8.12,19.20)	P = 0.5	16.06(9.69,26.27)	27.03(19.23,31.04)	11.41(8.12,19.20)	14.80(3.66,27.97)	P = 0.5
	**LF%**	41.61(29.41,54.38)	45.55(36.21,58.68)	P = 0.07	38.34(23.62,43.83)	36.41(21.47,49.94)	45.55(36.21,58.68)	31.72(16.36,49.99)	P = 0.1
	**LF/HF**	2.66(1.74,6.28)	4.09(2.37,7.51)	P = 0.09	1.86(1.59,3.50)	1.01(0.39,2.02)	4.09(2.37,7.51)	2.37(1.03,5.70)	P = 0.1
	**LZC (3,0.001)**	0.91(0.86,0.95)	0.91(0.88,0.94)	P = 0.08	0.93(0.85,0.96)	0.88(0.79,0.97)	0.91(0.88,0.94)	0.87(0.76,0.92)	P = 0.2
**Entire HD**									
	**SDANN (ms)**	**44(31,62)**	**28(20,42)**	**P = 0.002**	42(30,61)§	24(21,27)	**46(30,61)§**	**31(18,45)**	**P = 0.004**
	**VLF (ms**^**2**^**)**	675(369, 1627)	415(207,1026)	P = 0.09	417(287, 697)	266(61,580)	972(411, 1851)	452(252,1160)	P = 0.5
	**HF%**	16.0(7.0,21.6)	16.9(9.5,29.1)	P = 0.3	20.9(13.5, 23.8)	23.0(19.3,33.9)	12.7(6.8,18.1)	14.7(6.9,29.1)	P = 0.1
	**LF%**	37.88(23.20,53.31)	38.52(22.30,54.50)	P = 0.3	37.09(23.20,46.30)	36.99(23.20,44.85)	38.52(22.30,54.50)	30.94(21.45,47.65)	P = 0.7
	**LF/HF**	3.07(1.55,5.13)	3.41(1.77,5.30)	P = 0.09	2.04(1.15,3.88)	1.18(0.56,1.99)	3.41(1.77,5.30)	2.57(0.90,4.79)	P = 0.6
	**LZC (3,0.001)**	0.90(0.86,0.93)	0.86(0.78,0.92)	P = 0.1	0.90(0.88,0.91)	0.91(0.78,0.95)	0.90(0.86, 0.94)	0.85(0.78,0.91)	P = 0.3

Values of SDANN, VLF, HF% and LZC estimated during the first 30 min of the HD session and for the entire HD session. The values are expressed as median (25°, 75° percentiles). Significant values are indicated in bold.

L group: FO_pre_ ≤ 2.5 L; H group: FO_pre_ > 2.5 L; d: diabetic; nd: non diabetic.

Post-hoc comparisons between the four subgroups (Ld vs Lnd, Hd vs Hnd): § P = 0.02 * P = 0.001.

**Figure 3:**
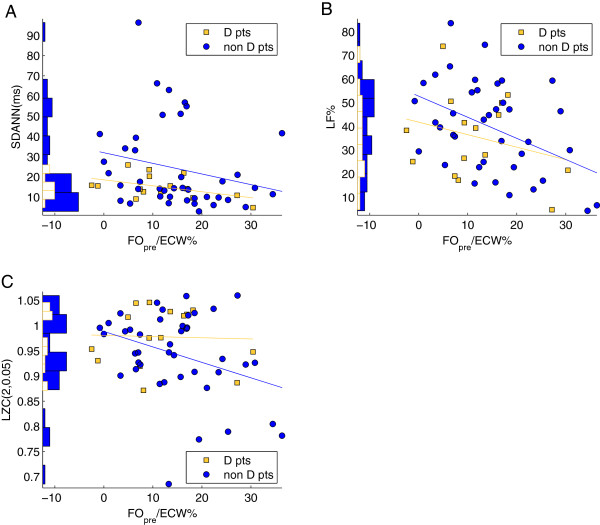
**Diabetes stratified results for FO/ECW% correlations with HRV indices.** Diabetes stratified results for FO/ECW% correlations with SDANN, LF% and LZC (2,0.05) indices estimated during the last 30 min of HD treatment (panel **A**, **B** and **C** respectively). Regression lines are portrayed for diabetic patients (D pts) and non-diabetic patients (non D pts) separately. Statistically significant correlations were only obtained for non-diabetic patients and the correlation coefficients of SDANN, LF% and LZC (2,0.05) indices were *r* = –0.32, *r* = –0.43 and *r* = –0.34, respectively. The histograms of HRV parameter distributions are shown on the left of each panel.

Regarding differences due to diabetic status, statistically significant correlations were only observed with non-diabetic patients. SDANN in non-diabetic subjects was significantly correlated with FO_pre_/ECW% for all time periods (during first 30 min of HD: *r* = -0.48, p-value = 0.001; during last 30 min of HD: *r* = -0.32, p-value = 0.04; during entire HD: *r* = -0.33, p-value = 0.03) Moreover, LF%, LZC(2,0.01) and LZC(2,0.005), estimated during the last 30 min of HD, were inversely correlated with FO_pre_/ECW% (LF%: *r* = -0.43, p-value = 0.006; LZC(2,0.01): *r* = -0.35, p-value = 0.03; and LZC(2,0.005): *r* = -0.34, p-value = 0.03) (Figure 3).

### Post-study follow-up

Five non-diabetic patients with persistent FO > 3 L were enrolled in the 3-month follow up program (Table 4). FO decreased significantly both after one month and after three months compared to baseline (BL: 3.78 ± 0.76 L, 1 M: 2.54 ± 0.49 L, 3 M: 2.23 ± 0.64 L, one-way ANOVA for repeated measurements p-value = 0.001, paired t-test with Bonferroni correction p-value = 0.02 between BL and 1 M, and between BL and 3 M values). Diastolic Blood Pressure (DBP) decreased after 3 months from the beginning of the study (BL: 79.9 ± 9.4 mmHg, 3 M: 71.2 ± 9.1 mmHg, one-way ANOVA for repeated measurements p-value = 0.06), while changes in Systolic Blood Pressure were not significant (BL: 153.4 ± 8.4 mmHg, 3 M: 146.6 ± 15.9 mmHg).

**Table 4:** Data and comorbidities of the five patients followed up for 3 months post-study

**Patient ID**	**Sex**	**Vintage**	**Age**	**Diabetes**	**LVH**	**PVD**	**CHF**	**CAD**	**Arrhythmias**
**223**	M	6	84	N	Y	Y	N	N	N
**2400**	F	6	45	N	Y	N	N	N	N
**1318**	M	13	73	N	N	N	N	N	Y
**6794**	M	1	72	N	N	N	N	N	N
**1400**	F	8	47	N	Y	N	Y	N	Y

N = no Y = yes; LVH: left ventricular hypertrophy; PVD: peripheral vascular disease; CHF: congestive heart failure; CAD: coronary artery disease.

SDANN values for the entire HD session increased in all 5 patients (paired Wilcoxon signed test p-value = 0.06). LF and LF% assessed during the HD session decreased in 4 out 5 patients, in particular, in patients 223 and 2400 with the highest baseline values (Table 5). Only one patient (ID = 6794) displayed a different trend as he was characterized by very low values of LF and LF% at baseline, but after 3 months LF and LF% reached similar values to the other patients.

**Table 5:** HRV values obtained during the follow-up study

	**LF (ms**^**2**^**)**			**LF%**			**SDANN (ms)**		
**Patient ID**	**BL**	**1 M**	**3 M**	**BL**	**1 M**	**3 M**	**BL**	**1 M**	**3 M**
**223**	1033.6	271	245.4	55.6	52.9	38.4	47.3	29	57
**2400**	2938	1101.5	284.3	68.4	47.1	36.3	48.5	70.3	68.7
**1318**	955.3	622.9	730.5	51.3	43.9	47.9	27.5	29.8	31.6
**6794**	6.7	227.4	49.5	1.9	39	20.1	34.8	31.1	101.8
**1400**	151.6	144	82.3	28.2	39	23.8	32.7	91.4	157.9
	955.3(115.4,1509.7)	271.0(206.6,742.5)	245.4(74.1,395.9)	51.3(21.6,58.8)	43.9(39.0,48.5)	36.3(22.9,40.8)	34.8(31.4,47.6)	31.1(29.6,75.6)	68.7(50.6,115.8)*

SDANN, LF component and LF% indexes measured during the HD session are shown for each patient at the start (baseline, BL), after 1 month (1 M), when the hydration target was planned to be reached, and after 3 months (3 M). The last row shows the values of median (25°, 75° percentiles).

*Paired Wilcoxon signed test p-value = 0.06 (3 M vs. BL).

## Discussion

FO and alterations in the ANS are associated with increased risk of cardiovascular mortality in CKD 5 patients. To the best of our knowledge, this is the first study in patients on chronic HD to quantify the relationship between fluid status and ANS, as assessed by HRV.

Several studies have shown that time- or frequency-domain measures of HRV may predict mortality and nonfatal arrhythmic events in patients with coronary heart disease [26,27]. Moreover, a reduced HRV has been consistently observed in patients with cardiac failure as well [28,29].

A significant increase in SDANN index, which reflects an increase in HRV, has been described in patients with moderate to severe heart failure after cardiac resynchronization therapy [30]. Noteworthy, lack of increase in SDANN four weeks after resynchronization therapy identified patients at highest risk for major cardiovascular events and no significant improvement in left ventricular ejection fraction, on the other hand, an increase of SDANN was associated with short-term improvements of functional heart capacity and of ANS modulation [30]. A decrease in complexity indices of HRV was associated as well with cardiovascular disease [31,32].

Large prospective ESRD cohort studies are lacking [33], and the results presented in this work can be compared mainly with data from studies on cardiovascular populations. In fact, the studies on HRV in ESRD patients are mainly focused on cardiac death stratification [34], to compare unstable and stable patients on HD [35,36] or to compare different type of treatment [37]. All these studies showed that a reduced HRV is an independent prognostic value of mortality and that HRV analysis permits to identify alterations in autonomic control, such in unstable patients. Furthermore, a recent study showed the midterm effects of initiation of chronic HD therapy and reported a significant improvement on HRV indices, such as SDANN and SDNN indices in chronic kidney disease (CKD) patients not affected by diabetes [38], nonetheless these indices were estimated over entire 24 h Holter recording and no information about hydration status was considered.

In this study we observed a significant correlation between HRV indices and FO. In particular, SDANN, VLF and LZC indices, which are related to ANS modulation, significantly correlated with FO when computed during whole dialysis session. These results support the hypothesis that FO may be associated with reduced HRV, likely because of heart functional changes induced by FO. Moreover, FO correlated positively with HF% spectral power during the whole treatment and negatively with LF during the last 30 minutes of the session. HF power reflects vagal and respiratory mediated changes in heart rate, whereas LF power is mostly a marker of sympathetic modulation [13]. According to these results, FO may be associated with reduction in the low oscillatory frequency, i.e. a reduced sympathetic response to the HD session. LF% index, which is related to the sympathetic activation, was inversely correlated to FO in the last 30 minutes of the treatment (Table 2, Figure 3B). Sympathetic response to HD is related to the fluid status of the patient [15], and it involves both the activation of baroreceptor reflex and peripheral vasoconstriction. A reduction of circulating blood volume, caused by the ultrafiltration process, produces a reduction of arterial transmural pressure and thus of peripheral vascular resistance (PVR), which may be sensed by arterial baroreceptors and trigger sympathetic outflow. The lower the FO, the higher the sympathetic autonomic response should be to maintain the arterial blood pressure.

Patients were divided in two groups according to their hydration state, using 2.5 L as the threshold [20,21]. At various times during treatment, SDANN, and VLF were lower in patients of group H (FO > 2.5 L) compared to group L (FO < 2.5 L), while HF% was instead higher in the H group compared to the L group (Table 3). There was also a trend towards lower LF% measured during the last 30 minutes of HD for the H group versus the L group. These results are in line with the hypothesis that FO may be associated with decreased HRV, and a higher parasympathetic activation, which may prevail on sympathetic response to HD session.

The vintage of group H patient was significantly longer than in group L. The effect of vintage on overhydration wasn’t yet investigated, but recently the work of Lindberger et al. [39] showed that the cognitive profiles seem to have a key role in the fluid control in patients on HD independently from the dialysis history. Therefore vintage is supposed not to be a determinant of the overhydration.

In patients with diabetic neuropathy, a reduction of HRV time-domain parameters has been demonstrated to be not only of negative prognostic value, but also to precede clinical signs of autonomic dysfunction [40,41]. Hence, patients were also divided in two groups according to diabetes mellitus status. In non-diabetic subjects, HRV indices significantly correlated with FO, as observed previously for the whole population. No such correlations were observed for patients with diabetes. This can be explained by diabetic neuropathy, which *per se* affects the autonomic nervous system [40], thus masking possible effects of FO. In fact, the effect of diabetes is a reduction of the absolute power of LF and HF, as observed in the general population [42].

In the post-study follow up, the FO of five non-diabetic patients having a predialysis FO greater than 3 liters was gradually reduced over one month, and then maintained over the two following months. After this strict control of fluid, an increase in SDANN index was observed in all patients, indicating an increase in HRV and an improvement in autonomic heart rate control. Moreover, an important decrease in LF and LF% was observed in four patients, and this was more pronounced in patients with higher baseline values. Furthermore, three out of these four patients were affected by left ventricular hypertrophy, which is known to be associated to sympathetic overactivity [43]. FO reduction was hypothesized to a positive effect on cardiac hypertrophy and a blunting effect on the sympathetic nervous system. Indeed, recent results have shown that a central inhibition of the sympathetic ANS rapidly reverses cardiac hypertrophy and problems associated with primary LV relaxation [43,44]. In one patient, LF and LF% did not decrease with the reduction of FO. This patient presented very low baseline values of LF and LF%, and this maybe related to the absence of LVH, to several arrhythmic events during hemodialysis and a slight bradycardia (~58 bpm).

Blood pressure control improved with the progressive reduction of fluid overload, as reflected in the reduction in DBP after 3 months of strict fluid management. The reduction of fluid overload and the associated decrease in sympathetic overactivity may have also stimulated a reduction in peripheral vascular resistance [43].

The obtained results and the observations collected in the post study follow-up reinforce the opinion that chronic volume overload is an important parameter to be carefully monitored as it is associated to mortality risk and to alteration of ANS, such sympathetic overactivity and low HRV. The effect of normalizing FO on ANS can be evaluated by means of a low cost examination, such as an ECG Holter. As a matter of fact a more appropriate management of fluid withdrawal during dialysis can translate in a better prognosis [45].

### Study limitation

The sample size didn’t permit to consider all the possible confounding factors, such as types of drugs. The comorbidities can represent another important confounding effect too. Only a subset of indices resulted significant, further studies needed to verify if this is due to different cofounding factors or these indices are the only ones associated to HD response. Finally, the post study follow up was limited to 5 patients only, and these findings must be considered explorative only.

## Conclusions

Our results demonstrated for the first time a correlation between HRV indices and FO: reduced HRV was associated with higher values of FO. A reduction of FO seems to positively affect sympathetic ANS activity and HRV in a small group of patients starting with persistent and clear FO. Further studies on a larger population and with a longer follow up are needed to confirm these preliminary findings and to determine the long-term effects of these modifications.

## Abbreviations

ANS: Autonomic nervous system; CKD: Chronic kidney disease; ECW: Extracellular water; ESRD: End stage renal disease; FO: Fluid overload; HD: Hemodialysis; HR: Heart rate; HRV: Heart rate variability; LVH: Left ventricular hypertrophy; PVR: Peripheral vascular resistance; SBP: Systolic blood pressure; DBP: Diastolic blood pressure; LF: Low frequency; HF: High frequency; TAFO: Time averaged fluid overload.

## Competing interests

The authors declare that they have no competing interests.

## Authors’ contributions

UM, FG, DNC and CT initiated and designed the study. FG, AC and AB collected data. MF, UM, AC, SC and CR analyzed and interpreted the data. MF, UM, AC, FG and DNC contributed to the writing of the manuscript. EG, MGS, SC and CR gave scientific advice in their field of expertise. All authors revised the manuscript and contributed to its improvement. All authors read and approved the final munuscript.

## Pre-publication history

The pre-publication history for this paper can be accessed here:

http://www.biomedcentral.com/1471-2369/15/26/prepub
